# Constructing treatment selection rules based on an estimated treatment effect function: different approaches to take stochastic uncertainty into account have a substantial effect on performance

**DOI:** 10.1186/s12874-019-0805-x

**Published:** 2019-08-01

**Authors:** Maren Eckert, Werner Vach

**Affiliations:** 1 Institute of Medical Biometry and Statistics, Section of Health Care Research and Rehabilitation Research, Faculty of Medicine and Medical Center - University of Freiburg, Hebelstr. 11, Freiburg, 79104 Germany; 2 Department of Orthopaedics and Traumatology, University Hospital Basel, Spitalstr. 21, Basel, CH-4031 Switzerland

**Keywords:** Interaction models, Pointwise confidence band, Root, Simultaneous confidence band, Treatment selection

## Abstract

**Background:**

Today we are often interested in the predictive value of a continuous marker with respect to the expected difference in outcome between a new treatment and a standard treatment. We can investigate this in a randomized control trial, allowing us to assess interactions between treatment and marker and to construct a treatment selection rule. A first step is often to estimate the treatment effect as a function of the marker value. A variety of approaches have been suggested for the second step to define explicitly the rule to select the treatment, varying in the way to take uncertainty into account. Little is known about the merits of the different approaches.

**Methods:**

Four construction principles for the second step are compared. They are based on the root of the estimated function, on confidence intervals for the root, or on pointwise or simultaneous confidence bands. All of them have been used implicitly or explicitly in the literature. As performance characteristics we consider the probability to select at least some patients, the probability to classify patients with and without a benefit correctly, and the gain in expected outcome at the population level. These characteristics are investigated in a simulation study.

**Results:**

As to be expected confidence interval/band based approaches reduce the risk to select patients who do not benefit from the new treatment, but they tend to overlook patients who can benefit. Simply using positivity of the estimated treatment effect function for selection implies often a larger gain in expected outcome.

**Conclusions:**

The use of 95% confidence intervals/bands in constructing treatment selection rules is a rather conservative approach. There is a need for better construction principles for treatment selection rules aiming to maximize the gain in expected outcome at the population level. Choosing a confidence level of 80% may be a first step in this direction.

**Electronic supplementary material:**

The online version of this article (10.1186/s12874-019-0805-x) contains supplementary material, which is available to authorized users.

## Background

Today we are often confronted with the task to investigate the predictive value of a continuous marker with respect to the expected difference in outcome between a new treatment and a standard treatment. A randomized controlled trial (RCT) can (and should) be used for such an investigation. It does not only allow to demonstrate an interaction between treatment choice and the marker, but also to construct a treatment selection rule. Such a rule aims at identifying those patients who can expect to benefit from the new treatment. It is a function of the marker value and hence can be applied also to future patients outside of the trial.

Several statistical methods have been proposed in the literature to construct treatment selection rules. Many of them are based on estimating the treatment effect *θ*(*x*) as a continuous function of the biomarker value *x*. Both parametric [1–3] as well as semi- or nonparametric approaches [4–6] can be found. However, although estimating *θ*(*x*) is a valuable step, it does not automatically provide a rule to determine those biomarker values with *θ*(*x*)>0; it remains the question whether and how to take stochastic uncertainty of $\hat \theta (x)$ into account.

Confidence bands have been considered by several authors to describe the uncertainty in $\hat \theta (x)$. Pointwise bands (e.g. [5]) and simultaneous confidence bands (e.g. [4]) as well as both together (e.g. [7, 8]) have been suggested. Mackey and Bengtsson, Riddell et al. [1, 3] suggest to construct a confidence interval for the root of *θ*(*x*) (with respect to 0 or another threshold), and similarly [2] suggest to compute horizontal confidence intervals. In contrast, some authors (e.g. [6]) only present a raw estimate of *θ*(*x*). However, all these authors do not explicitly address the question how to move from a (graphical) illustration of uncertainty to a concrete rule.

In recent years, there are some papers addressing the question more explicitly. Baker and Bonetti [9] as well as [10] suggest to check where the lower bound of the simultaneous confidence interval of the estimated subgroup treatment effect is positive. The former uses a confidence level of 95% and the latter one of 99%. In an overview about the construction of treatment selection rules [11] also consider pointwise and simultaneous confidence bands and rules based on comparing the lower bound with 0 or another given threshold.

In summary, we would like to argue that all authors directly or implicitly suggest to use one of the following types of treatment selection rules: If only the estimate $\hat \theta (x)$ is (graphically) presented, in future all patients with $\hat \theta (x) >0$ should receive the new treatment. If pointwise or simultaneous confidence bands for the treatment effect are also shown, all covariate values *x* with positive values of the lower bound should define the treatment selection rule. If a confidence interval for the root of *θ*(*x*) is given, only x-values outside of this interval satisfying also $\hat \theta (x)>0$ define the patients to be selected for the new treatment. We focus in this paper on the threshold 0 for the treatment effect, but our considerations are also applicable for any other threshold.

It is the purpose of this paper to give some insights into the performance of these principles to construct treatment selection rules. We are interested in differences in the impact for future patients outside of the trial when following the various principles. As potential impact we consider the correct identification of patients who do or do not benefit from the new treatment and the change in outcome at the population level.

## Methods

### Notation

To compare these principles we introduce some basic notations. Let *X* be the continuous covariate representing the biomarker value. Let *Y* be a continuous outcome and *T* the treatment indicator, randomized with a 50 percent chance to 0 or 1, and indicating a treatment with the standard or the new treatment, respectively. The treatment effect *θ*(*x*) is defined as the difference between the expected outcomes:

$$\theta(x) := E (Y \mid X = x, T = 1) - E (Y\mid X=x, T=0) $$

We assume that higher values of *Y* represent a higher treatment success. Thus, a positive treatment effect characterizes superiority of the new treatment.

A treatment selection rule can be regarded as the choice of a subset *C* of all possible values of *X*. Patients with covariate values in *C* should receive the new treatment instead of the standard treatment in future. A construction method is an algorithm to transform the data (*Y*_*i*_,*X*_*i*_,*T*_*i*_)_*i*=1,...,*n*_ observed in an RCT into a set *C*. Since the result of a construction method depends on random data, we consider it as a set-valued random variable $\mathcal {C}$. We can study the performance of the construction method by considering the distribution of $\mathcal {C}$.

### Performance characteristics

We start by defining quality measures for a single set *C*. Since this set *C* determines the treatment selection for future patients, we introduce a new random variable *X*^∗^ denoting the biomarker value for future patients. We consider three quality measures:

$$\begin{array}{@{}rcl@{}} \text{Sensitivity} &:= & P (X^{*} \in C \mid \theta(X^{*}) \geq 0) \\ \text{Specificity} &:=& P (X^{*} \not \in C \mid \theta(X^{*}) < 0) \\ \textrm{Overall gain} &:=& E (\theta(X^{*}) {1} \mathrm{I}_ {X^{*} \in C}) \end{array} $$

Sensitivity and specificity focus on the correct classification of patients by the treatment selection rule. Sensitivity measures the ability to select those patients who can expect to benefit from the new treatment. Specificity measures the ability to avoid recommending the new treatment to patients who cannot benefit from it. The overall gain is a summary measure taking into account also the magnitude of the treatment effect. It represents the change in the average outcome (i.e. in *E*(*Y*)), when we apply the proposed treatment selection rule in future, i.e. patients with *x*^∗^∉*C* receive the standard treatment and patients with *x*^∗^∈*C* receive the new treatment. It takes into account that *θ*(*x*^∗^) may be actually negative for some patients selected by the rule. The gain can be also seen as one specific way to balance between sensitivity and specificity, or – to be precise – between true positive and false positive decisions. A patient with *θ*(*x*)>0 correctly selected to receive the new treatment gets a weight equal to his or her individual benefit. A patient with *θ*(*x*)<0 incorrectly selected to receive the new treatment gets a weight equal to his or her individual, negative benefit. All patients selected for standard treatment get a weight of 0.

We have chosen these three measures, as they cover important characteristics. The different construction principles mentioned in the introduction can be regarded as attempts to control the specificity at the price of a reduced sensitivity. The overall gain measures the success of obtaining a sufficient balance in the sense that a low specificity decreases the overall gain by including too many patients with a negative *θ*(*x*^∗^), and a low sensitivity decreases the overall gain by excluding too many patients with a positive *θ*(*x*^∗^). However, it takes also into account that it is most favourable to include patients with large positive values of *θ*(*x*^∗^) and least favourable to include patients with large negative values of *θ*(*x*^∗^). Measures similar to the overall gain have been considered in the literature, but mainly with respect to the optimal rule *C*={*x*∣*θ*(*x*)≥0} as a measure of the benefit we can expect from a new biomarker. See [2] and the references given there. In the presentation of the results we will also indicate the maximal possible overall gain as a benchmark, defined as $E (\theta (X^{*}) {1}\hspace {-.1cm} \mathrm {I}_{\theta (X^{*}) \geq 0})$.

To describe the performance of a construction method for treatment selection rules, we study the distribution of these three quality measures when applied to $\mathcal {C}$ under the assumption that *X*^∗^ follows the same distribution as *X*. In this paper we will only consider the mean of this distribution, i.e. the expected sensitivity, the expected specificity, and the expected overall gain. In the context of comparing different subgroup analysis strategies, the expected overall gain has also been considered by [12].

### Construction principles for treatment selection rules

As mentioned above, we will consider four different construction principles for the treatment selection rule. All of them are based on the assumption that we have some statistical method providing us with an estimate $\hat \theta (x)$. Three principles assume that we can also perform certain types of statistical inference in order to construct pointwise or simultaneous confidence bands of the treatment effect or confidence intervals for the roots of *θ*(*x*). In the sequel, let *l*_*p*_(*x*) and *l*_*s*_(*x*) denote the value of the lower bound of a 95 percent pointwise and simultaneous confidence band, respectively. Let CI(*x*_*r*_) denote a confidence interval around any root *x*_*r*_, i.e. $x_{r} \in \hat \theta ^{-1}(0) =\{x \mid \hat \theta (x)=0\}$. Then, the construction principles can be described like shown in Table 1.

**Table 1:** Construction principles and the corresponding treatment selection rules

Construction principle	Treatment selection rule
estimator (EST)	$\mathcal {C}_{\text {EST}} := \{ x \mid \hat \theta (x) \geq 0 \}$
95 percent pointwise confidence band (POI)	$\mathcal {C}_{\text {POI}} := \{ x \mid l_{p}(x) \geq 0 \}$
95 percent simultaneous confidence band (SIM)	$\mathcal {C}_{\text {SIM}} := \{ x \mid l_{s}(x) \geq 0 \}$
95 percent confidence interval of all roots (CIR)	$\mathcal {C}_{\text {CIR}} := \{ x \mid \hat \theta (x) \geq 0 \} \ \backslash \bigcup \limits _{x_{r} \in \theta ^{-1}(0)} \text {CI}(x_{r})$

There is a close conceptual relation between the two principles POI and CIR. Both aim at excluding marker values *x* for which *θ*(*x*)=0 is "likely". POI tries to identify these values by considering the uncertainty in $\hat \theta (x)$. CIR tries to identify these values by considering the uncertainty in determining the root(s) of *θ*(.). (There can be several roots when *θ*(.) is chosen as a non-linear function, resulting in the somewhat technical definition shown above). Moreover, there is a direct mathematical relation. If a pointwise 1−*γ* confidence band for *θ*(.) is given, we can interpret it not only vertically, but also horizontally in the following sense: If for a given *θ*_*t*_ we consider all values of *x* such that (*θ*_*t*_,*x*) is within the confidence band, then these values define a 1−*γ* confidence interval for *θ*^−1^(*θ*_*t*_). A proof is outlined in Additional file 1.

We will nevertheless consider POI and CIR as different approaches, as there are a variety of methods to obtain confidence intervals for *θ*^−1^(0). In particular we will consider a simple application of the delta rule to obtain standard errors of *θ*^−1^(0), as it has been also used in [1].

### Design of simulation study

**Fig. 1:**
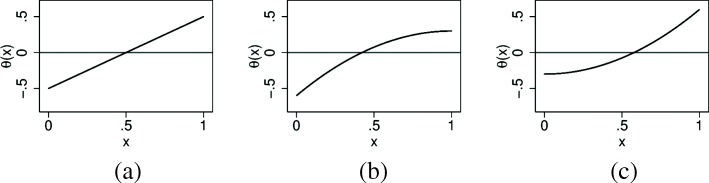
Three shapes for *θ*(*x*) with *β*=1. **a** *θ*(*x*)=*β*(*x*−0.5)**b** *θ*(*x*)=*β*(0.3−0.9(*x*−1)^2^)**c** *θ*(*x*)=*β*(−0.3+0.9*x*^2^)

In the general set up of the simulation study we generate a random variable *X*∈[0,1] representing the biomarker. *T* is generated as a Bernoulli random variable with a probability of 0.5. The continuous outcome *Y* follows a normal error model: *Y*=*α*(*X*)+*θ*(*X*)*T*+*ε*, where *ε*∼*N*(0,1). As the error variance is fixed to one, the value of *θ*(*x*) can be interpreted roughly as an effect size. We chose to investigate three shapes for the treatment effect function *θ*(*x*), a linear, a concave and a convex shape, see Fig. 1. Within each shape we have a scaling parameter *β* reflecting the steepness of the function. For the linear case we chose to investigate two different distributions of the biomarker, $X \sim \mathcal {U}(0,1)$ or $X \sim \mathcal {T}(0,1,1/3)$, while we only look at a uniformly distributed biomarker for the other two shapes. Here $\mathcal {T}(a,b,c)$ denotes a triangular distribution on the interval (*a,b*) with a mode in *c*. We do not consider the case of a normally distributed *X*, as the theory behind the methods we use to construct simultaneous confidence bands applies only to bounded intervals. Thus, in total we are investigating four scenarios summarized in Table 2. Without loss of generality we will assume *α*(*x*)=0 in generating the data. This is justified if we assume that the analysis models used are correctly specified with respect to *α*(*x*), such that the estimates for *θ*(*x*) are invariant under the transformations *Y*^′^=*Y*+*α*(*X*).

**Table 2:** Characteristics of the investigated scenarios

Scenario	Shape of *θ*(*x*)	Distribution of *X*
1	Linear: *θ*(*x*)=*β*(*x*−0.5)	$\mathcal {U}(0,1)$
2	Linear: *θ*(*x*)=*β*(*x*−0.5)	$\mathcal {T}(0,1,1/3)$
3	Concave: *θ*(*x*)=*β*(0.3−0.9(*x*−1)^2^)	$\mathcal {U}(0,1)$
4	Convex: *θ*(*x*)=*β*(−0.3+0.9*x*^2^)	$\mathcal {U}(0,1)$

In estimating *θ*(*x*) we use linear regression assuming a linear or a quadratic model for *α*(*X*) and *θ*(*X*):

$$\begin{aligned} \text{General analysis model: } & \quad Y=\alpha(X) + \theta_{\beta}(X)T\\ \text{Linear analysis model: } & \quad \alpha(X) = \alpha_{0} + \alpha_{1}X \\ & \quad\theta_{\beta}(X) = \beta_{0} + \beta_{1}X\\ \text{Quadratic analysis model: } & \quad \alpha(X) = \alpha_{0} + \alpha_{1}X \!+ \alpha_{2}X^{2}\\ &\quad \theta_{\beta}(X) = \beta_{0}\! +\! \beta_{1}X\! + \beta_{2}X^{2}\\ \end{aligned} $$

We will focus on using the “correct” analysis model, i.e. we apply the quadratic analysis model if *θ*(*x*) is concave or convex, and the linear model otherwise. The mathematics for building the pointwise and simultaneous confidence bands and the confidence intervals for the roots are outlined in Additional file 2. Candidate sets are constructed as described above for each of the four principles. However, this step is only performed in case of a significant interaction test, i.e. if *H*_0_:*β*_1_=0 or *H*_0_:*β*_1_=*β*_2_=0, respectively, could be rejected at the 5 percent level. In case of no significance all candidate sets are empty, i.e. $\mathcal {C} = \emptyset $.

In addition to the performance characteristics expected sensitivity, expected specificity, and expected overall gain, we also consider $P(\mathcal {C} \not = \emptyset)$, i.e. the probability to select at least some patients for the new treatment. We refer to this probability as the power, as it reflects the chance to get a “positive” result from the investigation of interest. It will also allow to judge the relevance of a chosen *β* value. The numerical computation of the performance characteristics is outlined in Additional file 3.

The sample size for a single trial was chosen in order to obtain for a clinically relevant situation a power of at least 90 percent with the most conservative method (i.e. SIM) in scenario 1. The relevant situation is characterized by one quarter of the patients to have a treatment effect above 0.2, corresponding to the choice *β*=0.8. The calculations resulted in a sample size of 1500, which we used for all scenarios. The number of repetitions in the simulation study was set to 2500, allowing to estimate a power of 90 percent with a standard error of 0.6 percent.

All calculations were performed using Stata 13. We used the available built-in procedures for generating random numbers, performing linear regression, construction of pointwise confidence bands (lincom) and application of the delta rule (nlcom). The calculation of the simultaneous confidence intervals were performed with self-written Stata programs and self-written functions in Mata, a programming language integrated in Stata. Source code for reproducing the results of the simulation can be viewed as Additional file 4 which also includes the data sets produced by the simulation.

## Results

### Scenario 1

**Fig. 2:**
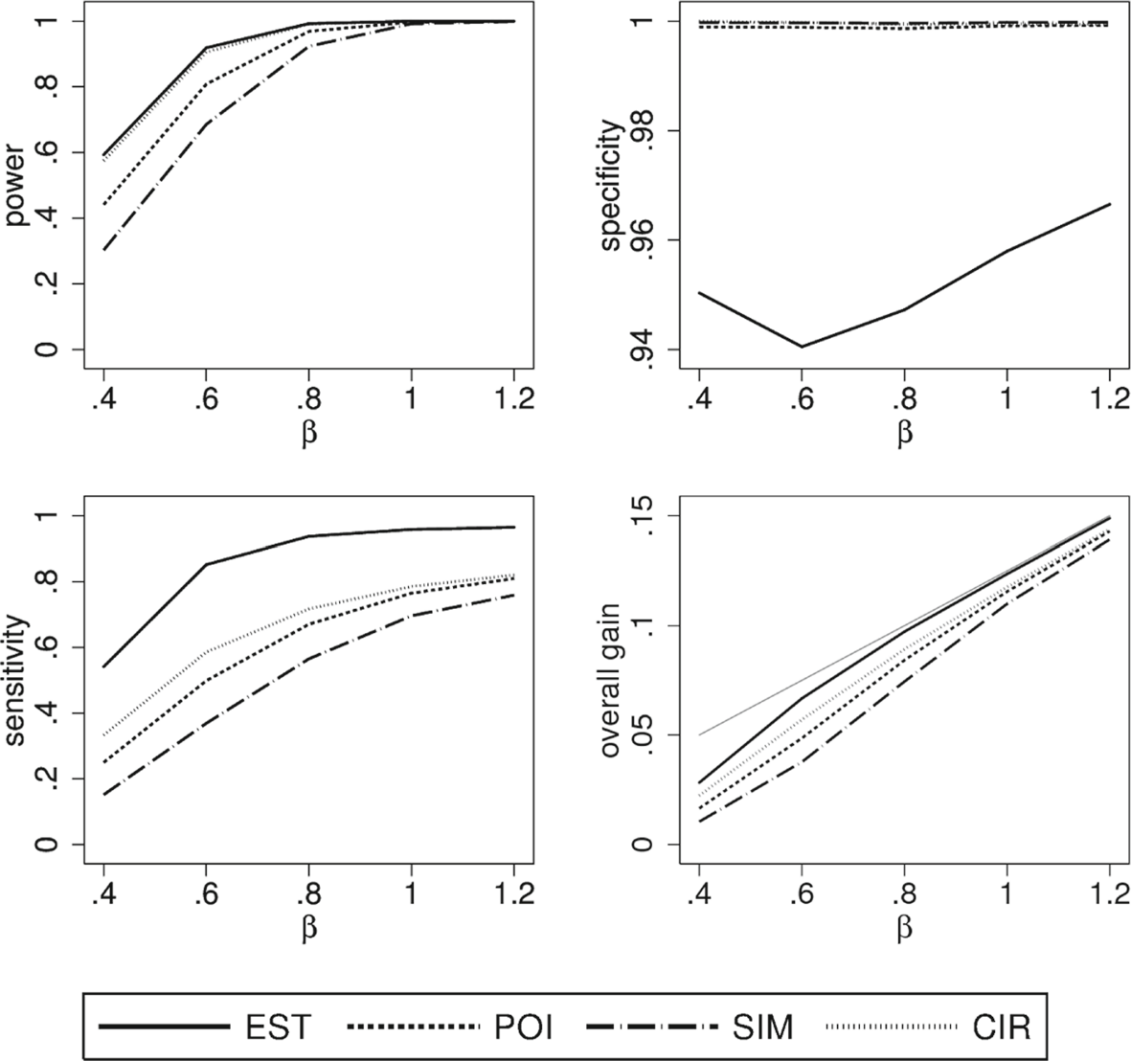
Simulation results of the performance characteristics for all four construction principles as function of *β*. Shown is scenario 1, i.e. *θ*(*x*) linear, $X \sim \mathcal {U}(0,1)$ using a linear model for analysis. For the overall gain, the thin grey line indicates the maximally possible overall gain

In this scenario we consider the case of a linear true treatment effect *θ*(*x*) and *X* being uniformly distributed. We can observe distinct differences between all four construction principles (Fig. 2). As expected EST has the highest power while SIM, as the most conservative method, has the lowest power. As *β* increases so does power, sensitivity and overall gain for all construction methods. In contrast, specificity is rather constant with a level of about 95 percent for EST and levels close to 100 percent for the other three methods. Sensitivity of POI, SIM, CIR is smaller compared to EST. SIM, being the most conservative method, evidently has the lowest value, while the most liberal method, EST, has the highest value. Looking at the overall gain and hence balancing the opposite trends for sensitivity and specificity, EST performed best and comes close to the maximal possible gain for *β*≥0.8. Using a confidence band or confidence interval to lower the number of patients incorrectly selected for the new treatment reduces the overall gain by a small amount.

### Scenario 2

**Fig. 3:**
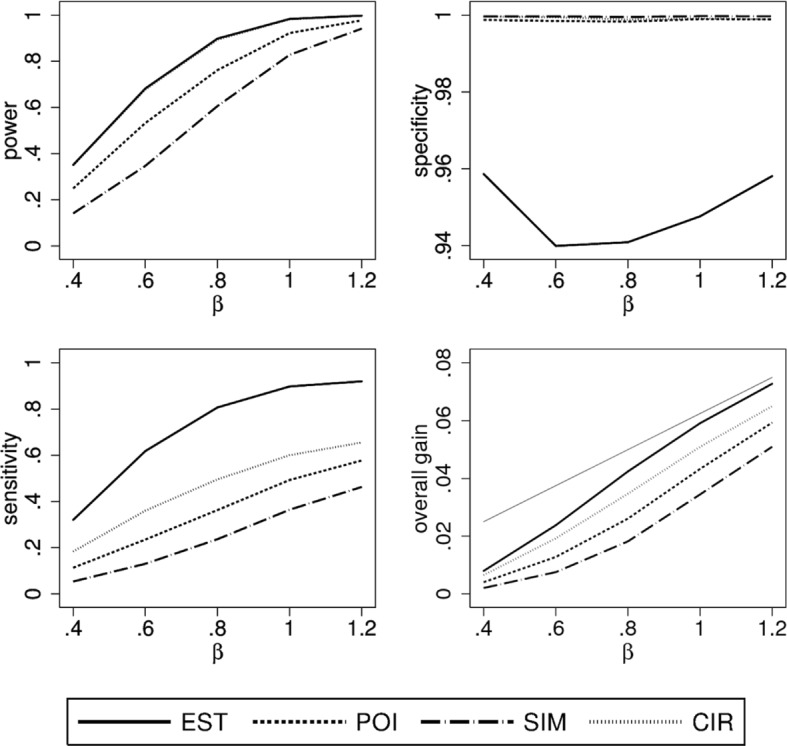
Simulation results of the performance characteristics for all four construction principles as function of *β*. Shown is scenario 2, i.e. *θ*(*x*) linear, $X \sim \mathcal {T}(0,1,1/3)$ using a linear model for analysis. For the overall gain, the thin grey line indicates the maximally possible overall gain

When changing the distribution of *X* to be triangular with mode at 1/3 there are less patients with a positive treatment effect. Power is lower in this situation (Fig. 3), as $\hat \theta (x)$ is more variable and confidence intervals for true positive effects are larger due to fewer observations. Specificity behaves similar as in scenario 1 but sensitivity and overall gain are considerably lower. Furthermore, there are bigger differences between the construction principles. For larger values of *β*, the loss in sensitivity is substantially greater when going from a liberal method to a more conservative one. A distinct loss can also be seen in the overall gain. For example, for *β*=0.8 more than half of the overall gain is lost when using SIM instead of EST and more than one third when using POI instead of EST. In contrast, the overall gain in EST is only about 15 percent below the maximal possible gain.

### Scenario 3

**Fig. 4:**
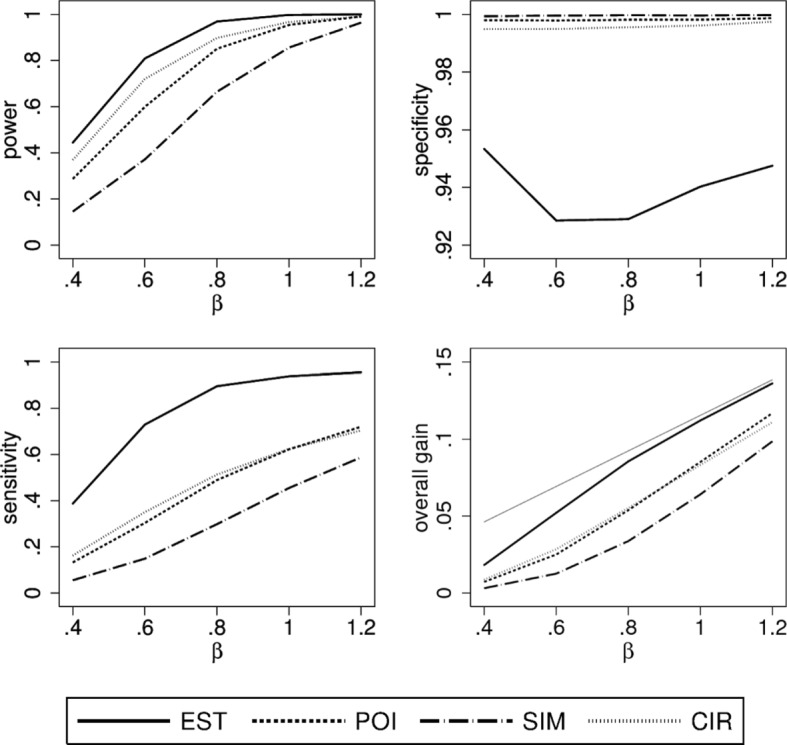
Simulation results of the performance characteristics for all four construction principles as function of *β*. Shown is scenario 3, i.e. *θ*(*x*) concave, $X \sim \mathcal {U}(0,1)$ using a quadratic model for analysis. For the overall gain, the thin grey line indicates the maximally possible overall gain

Figure 4 shows the results for this scenario with a uniformly distributed *X* and a concave true treatment effect. The results for power and specificity are similar to the first scenario but the specificity of EST is now slightly below 95 percent. On the other hand, there is a substantial loss in sensitivity and overall gain when comparing POI, SIM, and CIR with EST. This is probably due to the fact that the positive values of the treatment effect *θ*(*x*) are closer to zero than in the linear case (cf. Fig. 1). However, it still holds that the overall gain of EST is close to the maximal possible gain if *β*≥0.8.

### Scenario 4

**Fig. 5:**
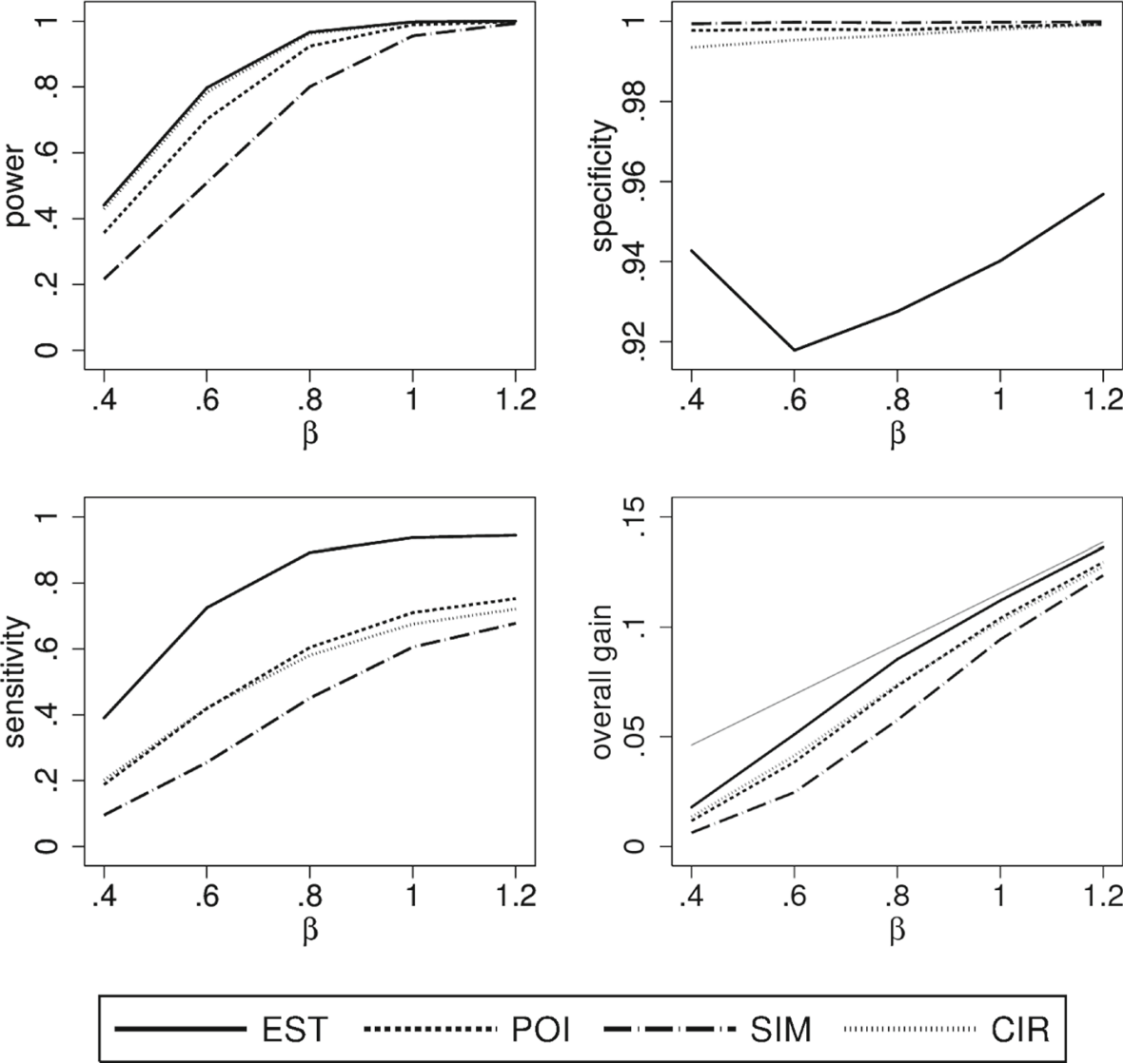
Simulation results of the performance characteristics for all four construction principles as function of *β*. Shown is scenario 4, i.e. *θ*(*x*) convex, $X \sim \mathcal {U}(0,1)$ using a quadratic model for analysis. For the overall gain, the thin grey line indicates the maximally possible overall gain

The last scenario considers a convex true treatment effect and a uniform distribution of *X*. The results shown in Fig. 5 look similar to the first scenario with a linear true treatment effect. The loss in sensitivity and overall gain is minor when choosing a more conservative method instead of EST, especially when compared to the last two scenarios. This can be explained by large positive values of *θ*(*x*) for the majority of patients with *θ*(*x*)≥0.

### Further results

When choosing the quadratic model for analysis in scenario 3 and 4, there may be a concern that the interaction test has little power as we test for a difference in two parameters. As we expect a monotone treatment effect it can be justified to use here also the interaction test based on the linear model. We investigated also this alternative, but the results were very similar. There may also be a concern that our results presented so far are too optimistic, as the model used to analyse the data coincides always with the true model. In Additional file 5 we present further results for misspecified models. They support the results presented so far.

Finally, we should mention that the performance characteristics between CIR and POI partially differed – in particular when using the linear analysis model – although POI can be also interpreted as a CIR approach. This indicates that using the delta method may not be very adequate. Indeed, in the linear analysis model the root is a ratio (cf. Additional file 2).

## Discussion

### Summary of results

The results of our simulation study indicate that using confidence bands for *θ*(*x*) or confidence intervals for *θ*^−1^(0) to construct treatment selection rules are rather conservative approaches when compared to selecting just those patients with a positive treatment effect estimate. They allow to move the rate of incorrect selections in patients not benefiting from the new treatment from about 5 percent to nearly 0 percent. But we have to pay the price to overlook a substantial fraction of patients who could benefit from the new treatment. Consequently, we often obtain a substantially lower overall gain than it would be possible when just requiring positive treatment effect estimates. Actually, this simple approach allows often to approach the maximally possible gain.

### Outlook

The step from modelling treatment effects as a function of a covariate to explicit construction of treatment selection rules has not yet been addressed systematically in the literature. The results of our simulation study suggest that requiring the lower bound of a 95 percent confidence interval for *θ*(*x*) to be above 0 is a very strict rule. At first sight such a rule may make sense, as in deciding whether to select patients with the biomarker value *x* for the new treatment, we control the probability of a type I error in these patients: If patients with this value do not benefit on average from the new treatment, the probability to select the new treatment is limited to 2.5 percent. This sounds similar to the traditional rationale in RCTs. However, in traditional RCTs we make a decision for a large patient population. Now we make a decision for a very small patient population, namely those with a specific covariate value. So it might not be surprising that the probability of a type II error, namely to overlook the benefit from the new treatment for this small population, is actually rather large.

**Fig. 6:**
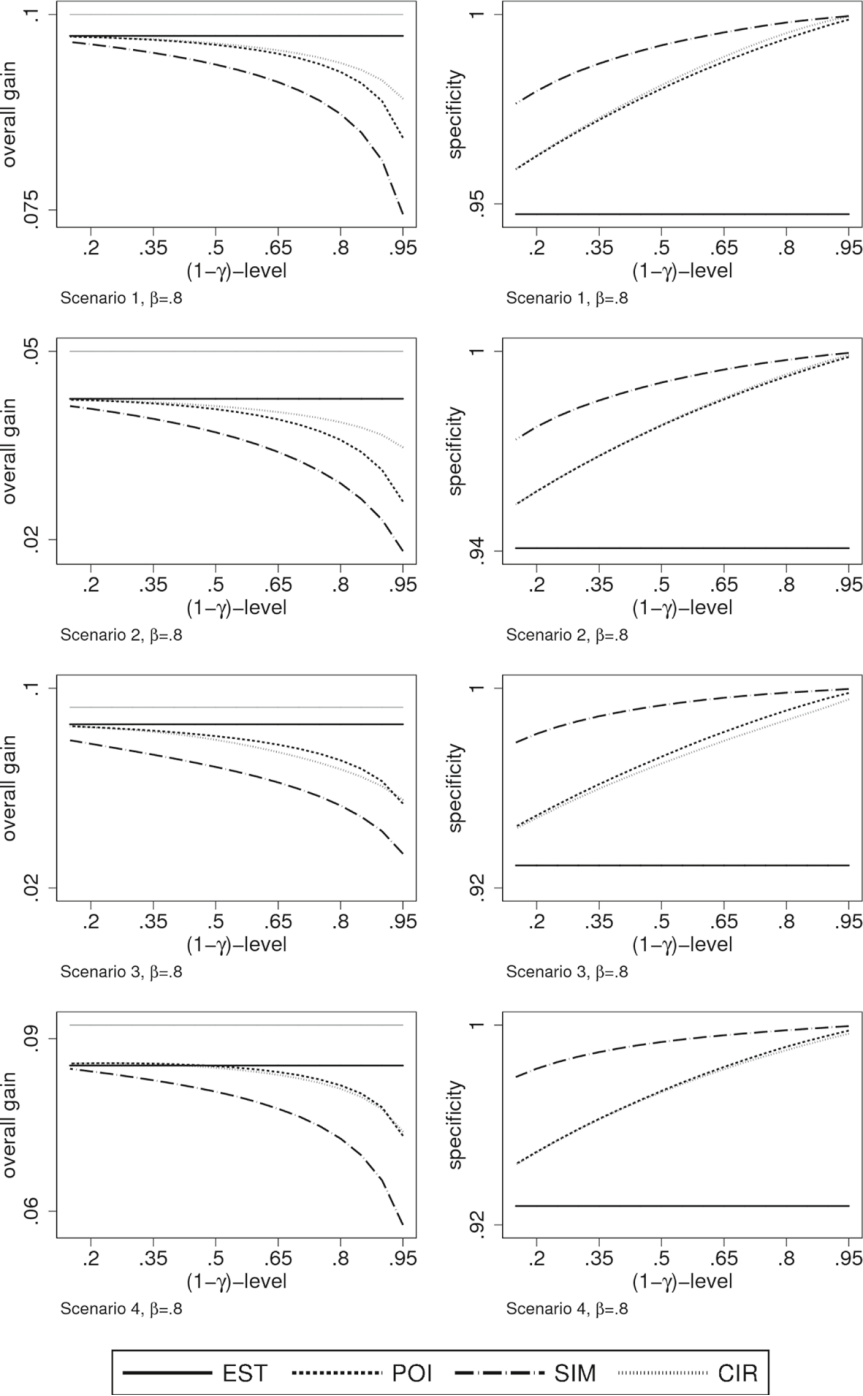
Simulation results of overall gain and specificity for *β*=0.8 in dependence of the (1−*γ*)-level. Shown are all four scenarios individually. The first two scenarios are analysed using a linear model, the latter two using a quadratic model. For the overall gain, the thin grey line indicates the maximally possible overall gain

Such considerations may suggest to allow higher type-I error rates in order to decrease the type II error rate and hence to improve the overall gain. In Fig. 6 we consider specificity and overall gain as a function of the (1- *γ*)-level of the confidence bands / the confidence interval in the case of *β*=0.8. We can observe a distinct increase of the overall gain when lowering (1−*γ*) from 0.95 to values around 0.8, but only a moderate decrease in specificity, keeping it at levels above 0.98 for all construction principles. This holds for all four scenarios and actually also for all values of *β*∈{.4,.6,.8,1,1.2}, see Additional file 6.

### Limitations

Our investigation was mainly limited to the case of correctly specified models in the sense that the true model is within the class of models used in the analysis. Misspecification of the model used for the analysis has a further impact on the performance characteristics, briefly touched in Additional file 5. However, the main point we tried to make in this paper is that even in the case of a correctly specified model, there is a need to come to a consensus on how to take uncertainty in parameter estimates into account when deriving a treatment selection rule. Consequently, our focus was also on rules varying in the way to take this uncertainty into account. Further variants of the rules which may take other aspects into account were not considered. For example rules of the type $\hat \theta (x)>c$ for some *c* may aim to take the clinical relevance of the treatment effect into account. We also focused on the three specific performance characteristics sensitivity, specificity and gain, as these were sufficient to make our point. However, for a complete picture it might be necessary to take further aspects into account, for example we can define the unmet gain as the average potential benefit for patients with *θ*(*x*)>0 who are overlooked by the rule.

Future comparisons should also include methods based on selecting optimal cutpoints directly, for example those on fitting cut point models [13, 14], or using the treatment selection curve [15]. Also alternatives to simply using an interaction test as pretest [2] can have an impact on the performance. In particular such alternatives may take into account the possibility that all patients may benefit from the new treatment to a similar degree.

## Conclusions

The use of 95% confidence intervals/bands in constructing treatment selection rules is a rather conservative approach. There is a need for better construction principles for treatment selection rules aiming to maximize the gain in expected outcome at the population level. Choosing a confidence level of 80% may be a first step in this direction.

## Additional files

Additional file 1 An equivalence between pointwise confidence bands for *θ*(.) and *θ*^−1^(.). (PDF 82 kb)

Additional file 2 Construction of the pointwise and simultaneous confidence bands and the confidence interval of the roots. (PDF 111 kb)

Additional file 3 Calculation of quality measures. (PDF 97 kb)

Additional file 4 Stata source code written for the simulation and data sets produced by simulation. (ZIP 42 kb)

Additional file 5 Results for the case of misspecified models. (PDF 236 kb)

Additional file 6 Simulation results for overall gain and specificity in dependence of the (1−*γ*)-level for various values of *β*. (PDF 492 kb)

## Data Availability

All Stata code used and data sets generated by the simulation are provided as Additional file 4.

## Abbreviations

CIR: Treatment selection rule using a 95 percent confidence interval of all roots
EST: Treatment selection rule using the estimator
POI: Treatment selection rule using a 95 percent pointwise confidence band
RCT: Randomized control trial
SIM: Treatment selection rule using a 95 percent simultaneous confidence band
