# Supplementary material for: Constructing treatment selection rules based on an estimated treatment effect function: different approaches to take stochastic uncertainty into account have a substantial effect on performance
Source: BMC Med Res Methodol. 2019 Aug 1;19:168. doi: 10.1186/s12874-019-0805-x (PMC6676573; doi:10.1186/s12874-019-0805-x)
Supplement: Supplementary file 1 — An equivalence between pointwise confidence bands for θ(.) and θ−1(.). (PDF 82 kb) [file 12874_2019_805_MOESM1_ESM.pdf]

Additional File 1: An equivalence between pointwise confidence bands for $\theta(.)$ and $\theta^{-1}(.)$

Let $\mathrm{CI}(x)$ denote a $1-\gamma$ pointwise confidence band for $\theta(x)$. For any η of the target space of $\theta(.)$ then $\varphi_{\eta,x} := \mathbb{1}(\eta \notin \mathrm{CI}(x))$ defines a level γ test for $H_0 : \theta(x) = \eta$ and we have $\mathrm{CI}(x) = \{\eta | \varphi_{\eta,x} = 0\}$. Now $\theta(x) = \eta$ is equivalent to $x \in \theta^{-1}(\eta)$. Consequently, $\varphi_{\eta,x}$ is a level γ test for $H_0 : x \in \theta^{-1}(\eta)$. Hence $\tilde{\mathrm{CI}}(\eta) := \{x \mid \varphi_{\eta,x} = 0\}$ is a confidence band for $\theta^{-1}(\eta)$ in the sense that for any $x \in \theta^{-1}(\eta)$ it holds that $P(x \in \tilde{\mathrm{CI}}(\eta)) \geq 1-\gamma$.
