# Supplementary material for: Constructing treatment selection rules based on an estimated treatment effect function: different approaches to take stochastic uncertainty into account have a substantial effect on performance
Source: BMC Med Res Methodol. 2019 Aug 1;19:168. doi: 10.1186/s12874-019-0805-x (PMC6676573; doi:10.1186/s12874-019-0805-x)
Supplement: Supplementary file 2 — Construction of the pointwise and simultaneous confidence bands and the confidence interval of the roots. (PDF 111 kb) [file 12874_2019_805_MOESM2_ESM.pdf]

Additional File 2: Construction of the pointwise and simultaneous confidence bands and the confidence interval of the roots

Pointwise confidence bands

A pointwise $1-\gamma$ confidence band $[l_p(x), u_p(x)]$ for $\theta(x)$ has to satisfy the property $P(\theta(x) \in [l_p(x), u_p(x)]) \geq 1-\gamma$ for each $x \in (0,1)$. It can be constructed using the fact that $\hat{\theta}(x)$ is a linear function of $\hat{\beta}$ and β is part of the regression parameter vector (α, β) of the models presented in Section 5. The design matrix $\boldsymbol{X}$ of these models can be expressed as

$$\boldsymbol{X} = \begin{pmatrix} \boldsymbol{X}_1 & \boldsymbol{X}_1 \\ \boldsymbol{X}_0 & 0 \end{pmatrix}$$

with

$$\boldsymbol{X}_0 = \begin{pmatrix} 1 & x_1 \\ \vdots & \vdots \\ 1 & x_{n_0} \end{pmatrix} \quad \text{or} \quad \boldsymbol{X}_0 = \begin{pmatrix} 1 & x_1 & x_1^2 \\ \vdots & \vdots & \vdots \\ 1 & x_{n_0} & x_{n_0}^2 \end{pmatrix},$$

where x_i is the covariate value of the i-th patient among the n_0 patients with $T=0$, and $\boldsymbol{X}_1$ is defined analogously with the covariate values of the patients with $T=1$. Following Liu (2011), we have

$$[l_p(x), u_p(x)] = [\boldsymbol{x}'\hat{\boldsymbol{\beta}} \pm t^{\gamma/2}_{n-2(p+1)}\hat{\sigma}\sqrt{\boldsymbol{x}'\boldsymbol{\Delta}\boldsymbol{x}}]$$

where p is the number of covariates, i.e. $p=1$ in the linear analysis model or $p=2$ in the quadratic analyis model, respectively, $\boldsymbol{x}=(1,x)$ or $\boldsymbol{x}=(1,x,x^2)$, $\hat{\boldsymbol{\beta}}=(\hat{\beta}_0,\hat{\beta}_1)$ or $\hat{\boldsymbol{\beta}}=(\hat{\beta}_0,\hat{\beta}_1,\hat{\beta}_2)$, $\boldsymbol{\Delta}=(\boldsymbol{X}_1\boldsymbol{X}_1')^{-1}-(\boldsymbol{X}_0\boldsymbol{X}_0')^{-1}$ and $\hat{\sigma}$ is the estimator of the standard deviation σ of the error variance, i.e. $\hat{\sigma}=\sqrt{\left\|\boldsymbol{Y}-\boldsymbol{X}(\hat{\boldsymbol{\alpha}},\hat{\boldsymbol{\beta}})\right\|^2/(n-2(p+1))}$, where $\boldsymbol{Y}=(y_1,...,y_n)$ with y_i being the outcome of the i-th patient when ordered in correspondence with the design matrix. t^{α}_n denotes the upper α-quantile of a t-distribution with n degrees of freedom.

Simultaneous confidence bands

A simultaneous $1-\gamma$ confidence band $[l_p(x), u_p(x)]$ for $\theta(x)$ has to satisfy the property $P(\theta(x) \in [l_p(x), u_p(x)]$ for all $x \in (0,1)) \geq 1-\gamma$. Simultaneous confidence bands can be constructed by blowing up the pointwise confidence bands until this property is reached. So the bands have the form

$$[l_s(x), u_s(x)] = [\boldsymbol{x}'\hat{\boldsymbol{\beta}} \pm c\hat{\sigma}\sqrt{\boldsymbol{x}'\boldsymbol{\Delta}\boldsymbol{x}}] \tag{1}$$

with some constant c which has to be chosen accordingly. Following Liu (2011), in the case of a linear analysis model c has to solve the equation

$$\gamma = \frac{\phi}{\pi}\left(1+\frac{c^2}{(n-2)}\right)^{-(n-2)/2} - \frac{2}{\pi}\int_0^{(\pi-\phi)/2}\left(1+\frac{c^2}{(n-2)\sin^2(\Theta+\phi/2)}\right)^{-(n-2)/2} d\Theta$$

where $\cos\phi = (1,0)'\boldsymbol{\Delta}(1,1)/\sqrt{(1,0)'\boldsymbol{\Delta}(1,0)(1,1)'\boldsymbol{\Delta}(1,1)}$. We solve the equation by numerically approximating the integral using the trapezoidal rule and determining the solution numerically by using the bisection method.

In the case of a quadratic analysis model, we follow Knafl et al. (1985) and consider a grid $(x_1, \ldots, x_{1000})$ of x-values on the interval (0,1). As each $\hat{\theta}(x)$ is a linear function of $\hat{\beta}$, we can compute the covariance matrix of $\hat{\theta}(x_1), \ldots, \hat{\theta}(x_{1000})$. We then determine c by generating 10,000 draws from the corresponding multivariate normal distribution and looking for the largest value of c such that (1) holds. Note that we can assume an arbitrary value of the mean values, i.e. of $\theta(x)$, in these computations.

Confidence intervals for the roots

We estimate the roots of $\theta(x)$ by considering $h_\eta(\hat{\beta}) := \theta_{\hat{\beta}}^{-1}(\eta)$ and determining the set $h_0(\hat{\beta})$. In the case of two roots, we can express h_0 as two single valued functions functions $h_0^1(\hat{\beta})$ and $h_0^2(\hat{\beta})$ of $\hat{\beta}$, such that $x_r^j = h_0^j(\hat{\beta})$; otherwise, h_0 is already single valued and we have $x_r = h_0(\hat{\beta})$. Since h_0^j and h_0 are continuous and differentiable in $\hat{\beta}$, we can use the delta rule to determine approximately the variance of the roots, and hence can construct confidence intervals assuming a normal distribution of the roots.

In the case of a linear analysis model, we have a single root $x_r = -\frac{\beta_0}{\beta_1}$. In the case of a quadratic analysis model, we have no root, if $\beta_1^2 - 4\beta_0\beta_2 < 0$, a single root $x_r = -\beta_1/2\beta_2$ if $\beta_1^2 - 4\beta_0\beta_2 = 0$, or two roots $x_r = (-\beta_1 \pm \sqrt{\beta_1^2 - 4\beta_0\beta_2})/2\beta_2$ otherwise. In the latter case we ignored roots outside of the interval (-0.25,1.25) in applying CIR, as such roots are often associated with wide CIs covering the whole interval (0,1).

References

Knafl, G., Sacks, J., Ylvisaker, D.: Confidence bands for regression-functions. Journal of the American Statistical Association **80**(391), 683–691 (1985)

Liu, W.: Simultaneous Inference in Regression. CRC Press, Boca Raton (2011)
