# Supplementary material for: Constructing treatment selection rules based on an estimated treatment effect function: different approaches to take stochastic uncertainty into account have a substantial effect on performance
Source: BMC Med Res Methodol. 2019 Aug 1;19:168. doi: 10.1186/s12874-019-0805-x (PMC6676573; doi:10.1186/s12874-019-0805-x)
Supplement: Supplementary file 3 — Calculation of quality measures. (PDF 97 kb) [file 12874_2019_805_MOESM3_ESM.pdf]

Additional File 3: Calculation of quality measures

Let f_{X^*} be the density function of X^*. Let $C_T = \{x \mid \theta(x) \geq 0\}$. Then we can express sensitivity, specificity and overall gain as

$$\begin{aligned}
\text{Sensitivity} =& P\Big(X^* \in C \mid \theta(X^*) \geq 0\Big) = P\Big(X^* \in C \cap \theta(X^* \geq 0)\Big)\Big/P\Big(\theta(X^* \geq 0)\Big) \\
=& \int\limits_{C \cap C_T} f_{X^*}(x)\,dx \Big/ \int\limits_{C_T} f_{X^*}(x)\,dx \\
\text{Specificity} =& P\Big(X^* \notin C \mid \theta(X^*) < 0\Big) = P\Big(X^* \notin C \cap \theta(X^*) < 0\Big)\Big/P\Big(\theta(X^*) < 0\Big) \\
=& \int\limits_{\overline{C} \cap \overline{C_T}} f_{X^*}(x)\,dx \Big/ \int\limits_{\overline{C_T}} f_{X^*}(x)\,dx \\
\text{Overall gain} =& E(\theta(X^*)\mathbb{1}_{X^* \in C}) = \int\limits_{C} \theta(x) f_{X^*}(x)\,dx
\end{aligned}$$

Since for all construction methods and all choices of $\theta(x)$ the sets C, C_T and their complements can be written as the union of a finite number of disjunct intervals, we need only to consider how to compute $\int\limits_{(a,b)} f_{X^*}(x)\,dx$ and $\int\limits_{(a,b)} \theta(x) f_{X^*}(x)\,dx$. For the special cases of $\theta(x)$ to be linear or quadratic, we can derive explicit formulas for these integrals; the corresponding formulas are shown in the following table:

	$\int\limits_{(a,b)} f_{X^*}(x)\,dx$	$\int\limits_{(a,b)} \theta(x) f_{X^*}(x)\,dx$
$X \sim U(0,1)$	$b - a$	
$\theta(x) = \beta(x - 0.5)$		$\beta/2((b^2 - a^2) - (b - a))$
$\theta(x) = \beta(l + m(x + n)^2)$		$\beta((l + mn^2)(b - a)$ $+mn(b^2 - a^2) + m/3(b^3 - a^3))$
$X \sim T(0, 1, 1/3)$		
$\theta(x) = \beta(x - 0.5)$		
case 1: $1/3 < a < b$	$3(b - a) - 3/2(b^2 - a^2)$	$\beta(-3/2(b - a) + 9/4(b^2 - a^2) - (b^3 - a^3))$
case 2: $a < b \leq 1/3$	$3(b^2 - a^2)$	$\beta(2(b^3 - a^3) - 3/2(b^2 - a^2))$
case 3: $a \leq 1/3 < b$	$3((1/9 - a^2) + (b - 1/3)$ $-1/2(b^2 - 1/9))$	$\beta(7/36 - 2a^3 + 3/2a^2 - 3/2b + 9/4b^2 - b^3)$
