# Supplementary material for: Constructing treatment selection rules based on an estimated treatment effect function: different approaches to take stochastic uncertainty into account have a substantial effect on performance
Source: BMC Med Res Methodol. 2019 Aug 1;19:168. doi: 10.1186/s12874-019-0805-x (PMC6676573; doi:10.1186/s12874-019-0805-x)
Supplement: Supplementary file 5 — Results for the case of misspecified models. (PDF 236 kb) [file 12874_2019_805_MOESM5_ESM.pdf]

Additional File 5: Results for the case of misspecified models

To address the impact in using misspecified models in estimating the treatment effect function, we reanalysed in a first step scenario 1 and 2, both of which have a true linear shape for the treatment effect function, now using a quadratic model for the analyses, i.e. a model more flexible than necessary. When comparing Figures 7 and 8 with Figures 2 and 3, that is to say the more flexible analysing models with the less flexible ones, we could observe a decrease in sensitivity, specificity, power and overall gain, but the differences between the four principles became actually more distinct.

In a second step, we reanalysed scenario 3 and 4, both of which have a true quadratic treatment effect function, now using a linear model for the analysis, i.e. a misspecified model. Comparing Figure 9 with Figure 4 , we can observe that using a linear model in the case of a concave relationship implies a slightly higher specificity, which we can explain by the fact that we obtain on average a root larger than the true one. Consequently, we should expect a decrease in sensitivity, but interestingly, this only holds for large values of β, and never for SIM. For the overall gain we observe a minor increase for all approaches except for EST in the case of large values of β. So sometimes the gain in precision by using a misspecified model seems to outperfrom the bias. However, for our considerations it is important that the qualitative differences between the approaches remain, although they become less distinct. Comparing Figure 10 with Figure 5 , we can observe that using a linear model in the case of a convex relationship implies a lower specificity, which we can explain by the fact that on average the estimated root is smaller than the true one. The same argument explains the marked increase in sensitivity. For the overall gain there is nearly no change. The qualitative differences between the approaches remain – at a less distinct level.

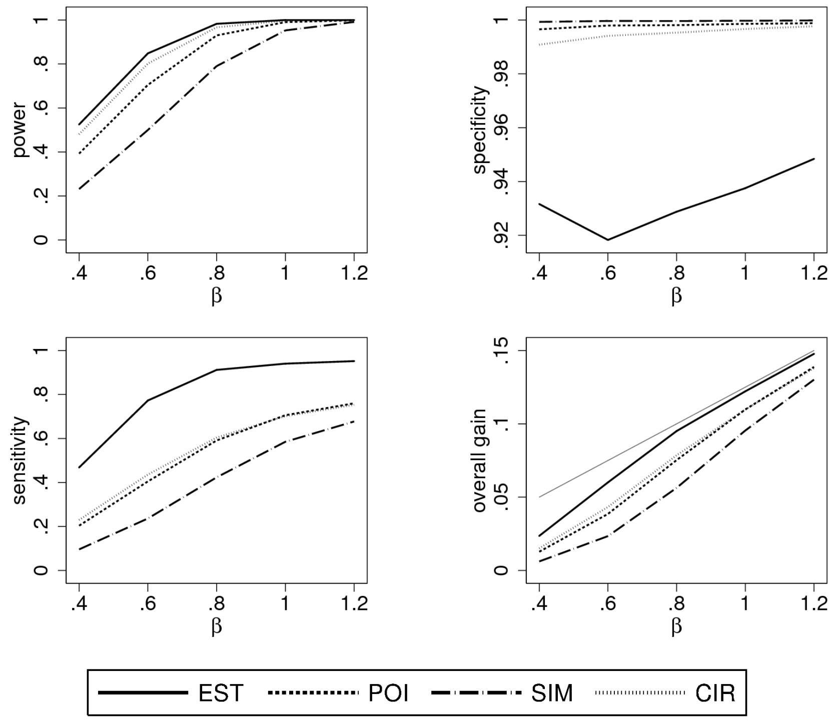

Figure 7 Simulation results, when using more flexible models for the analysis than necessary, of the performance characteristics for all four construction principles as function of β. Shown is scenario 1, i.e. $\theta(x)$ linear, $X \sim \mathcal{U}(0, 1)$ using a quadratic model for analysis. For the overall gain, the thin grey line indicates the maximally possible overall gain.

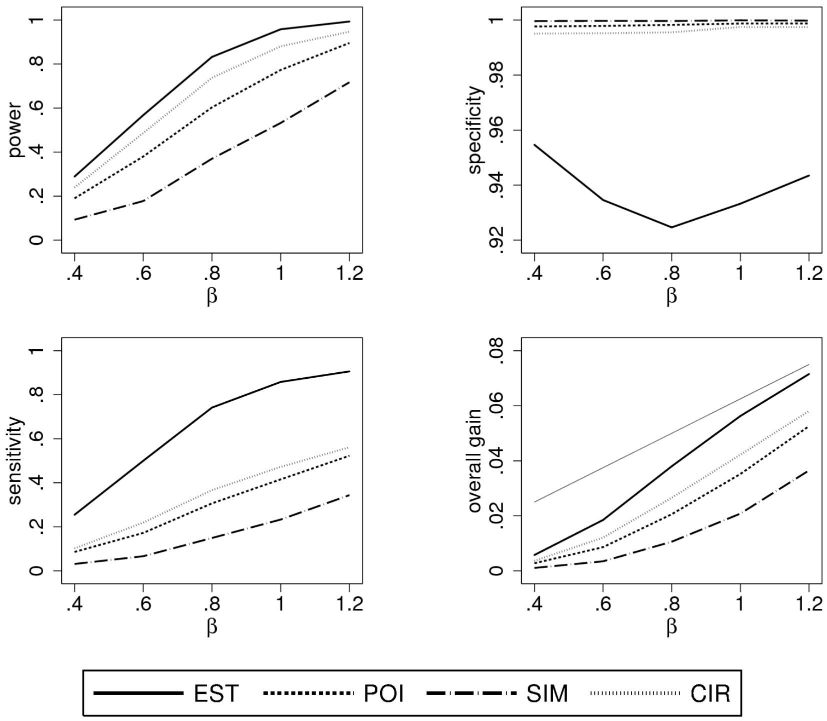

Figure 8 Simulation results, when using more flexible models for the analysis than necessary, of the performance characteristics for all four construction principles as function of β. Shown is scenario 2, i.e. $\theta(x)$ linear, $X \sim \mathcal{T}(0, 1, 1/3)$ using a quadratic model for analysis. For the overall gain, the thin grey line indicates the maximally possible overall gain.

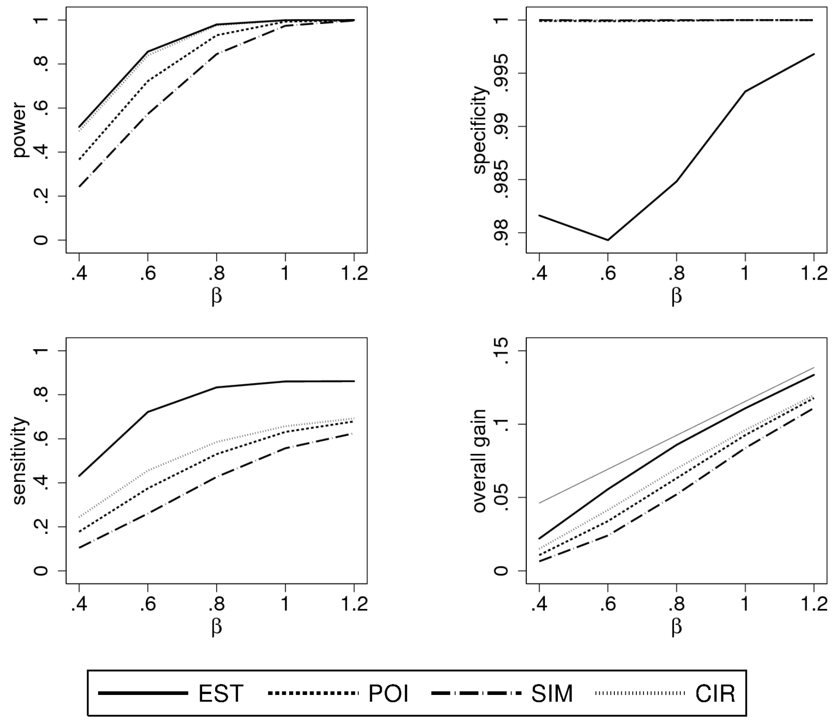

Figure 9 Simulation results, when using misspecified models for the analysis, of the performance characteristics for all four construction principles as function of β. Shown is scenario 3, i.e. $\theta(x)$ concave, $X \sim \mathcal{U}(0,1)$ using a linear model for analysis. For the overall gain, the thin grey line indicates the maximally possible overall gain.

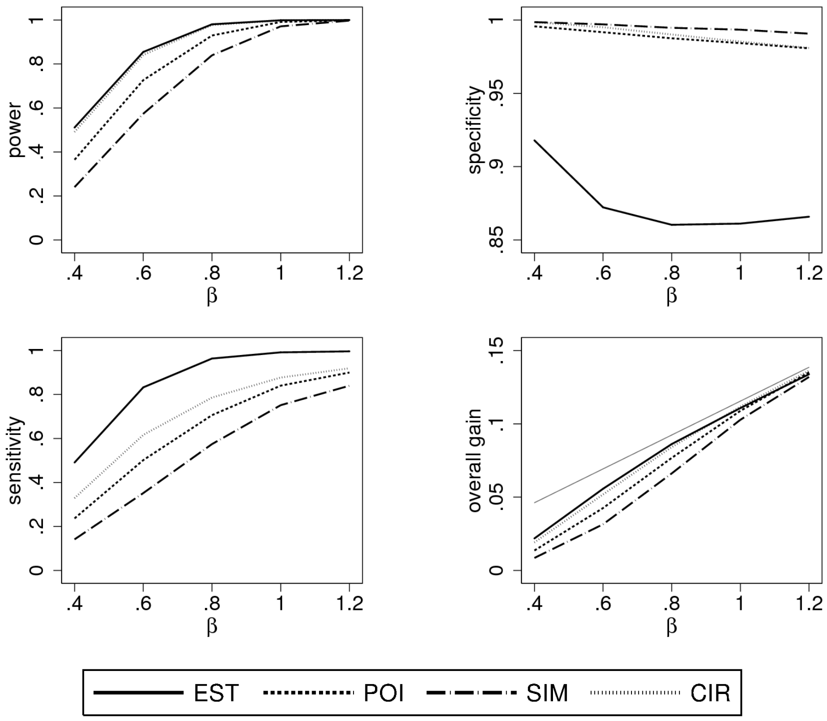

Figure 10 Simulation results, when using misspecified models for the analysis, of the performance characteristics for all four construction principles as function of β. Shown is scenario 4, i.e. $\theta(x)$ convex, $X \sim \mathcal{U}(0,1)$ using a linear model for analysis. For the overall gain, the thin grey line indicates the maximally possible overall gain.
