# Supplementary material for: Constructing treatment selection rules based on an estimated treatment effect function: different approaches to take stochastic uncertainty into account have a substantial effect on performance
Source: BMC Med Res Methodol. 2019 Aug 1;19:168. doi: 10.1186/s12874-019-0805-x (PMC6676573; doi:10.1186/s12874-019-0805-x)
Supplement: Supplementary file 6 — Simulation results for overall gain and specificity in dependence of the (1−γ)-level for various values of β. (PDF 492 kb) [file 12874_2019_805_MOESM6_ESM.pdf]

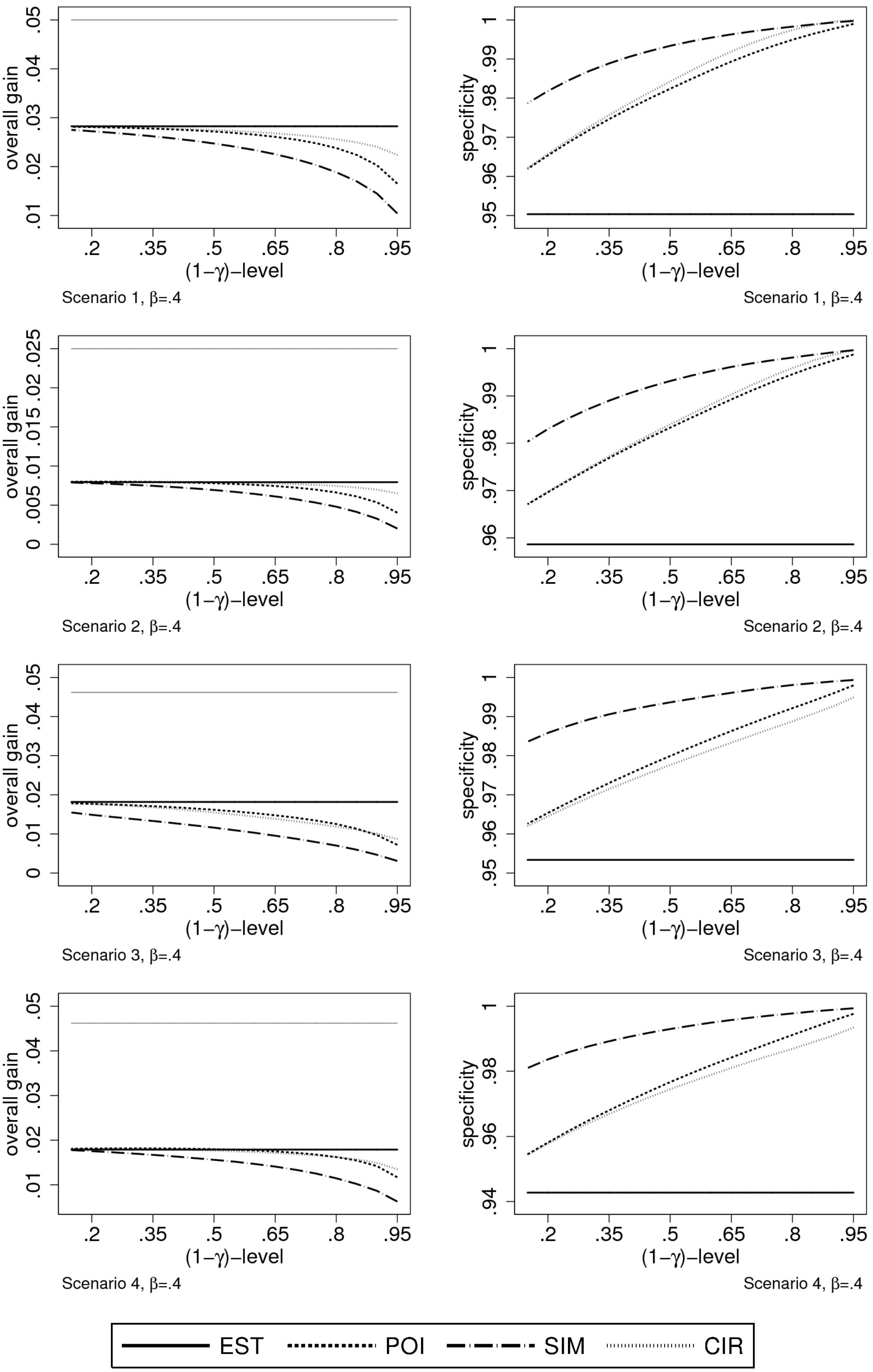

Figure Additional File 6a: Simulation results of overall gain and specificity for $\beta = 0.4$ in dependence of the $(1-\gamma)$-level. Shown are all four scenarios individually. The first two scenarios are analysed using a linear model, the latter two using a quadratic model. For the overall gain, the thin grey line indicates the maximally possible overall gain.

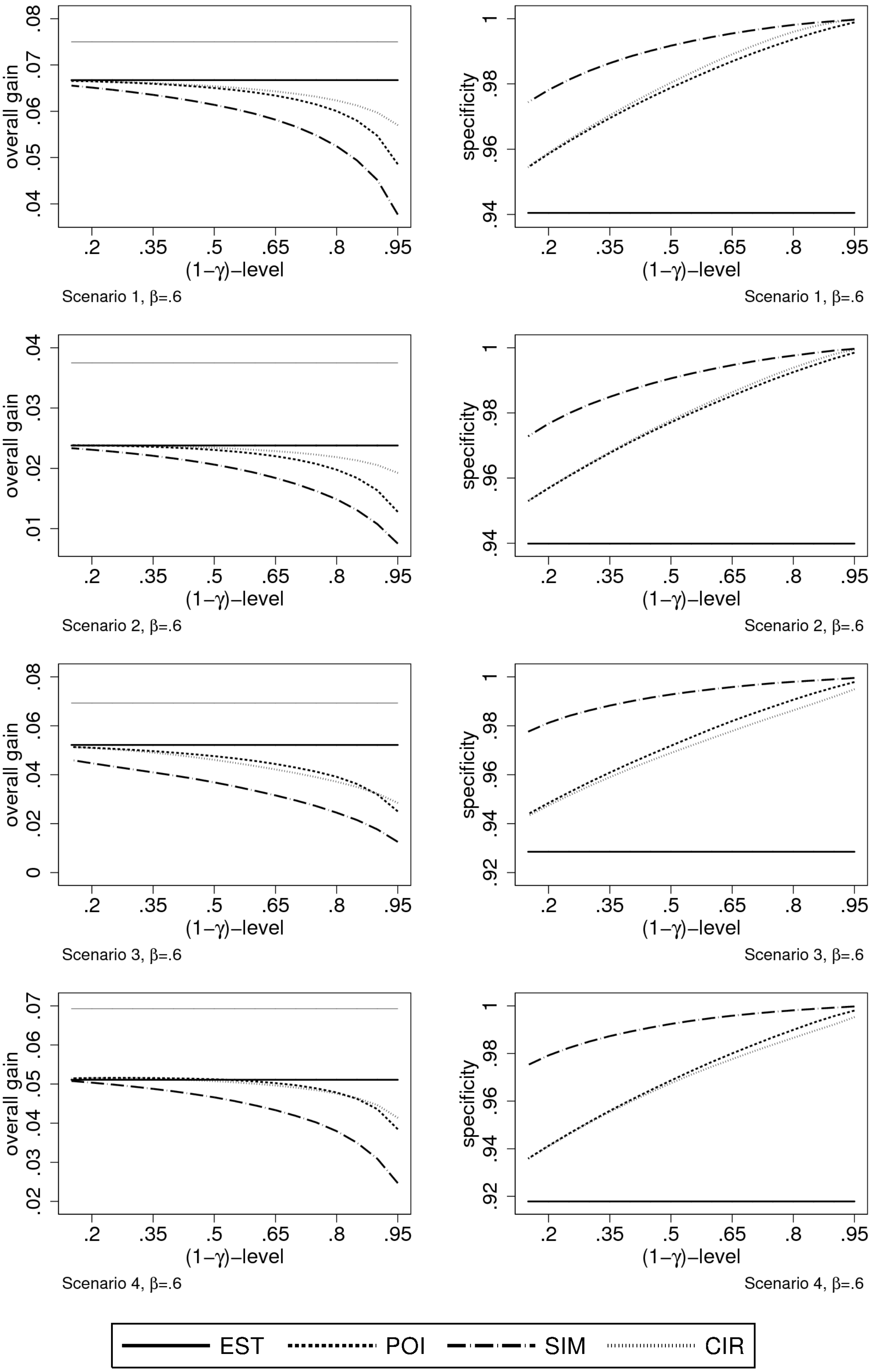

Figure Additional File 6b: Simulation results of overall gain and specificity for $\beta = 0.6$ in dependence of the $(1-\gamma)$-level. Shown are all four scenarios individually. The first two scenarios are analysed using a linear model, the latter two using a quadratic model. For the overall gain, the thin grey line indicates the maximally possible overall gain.

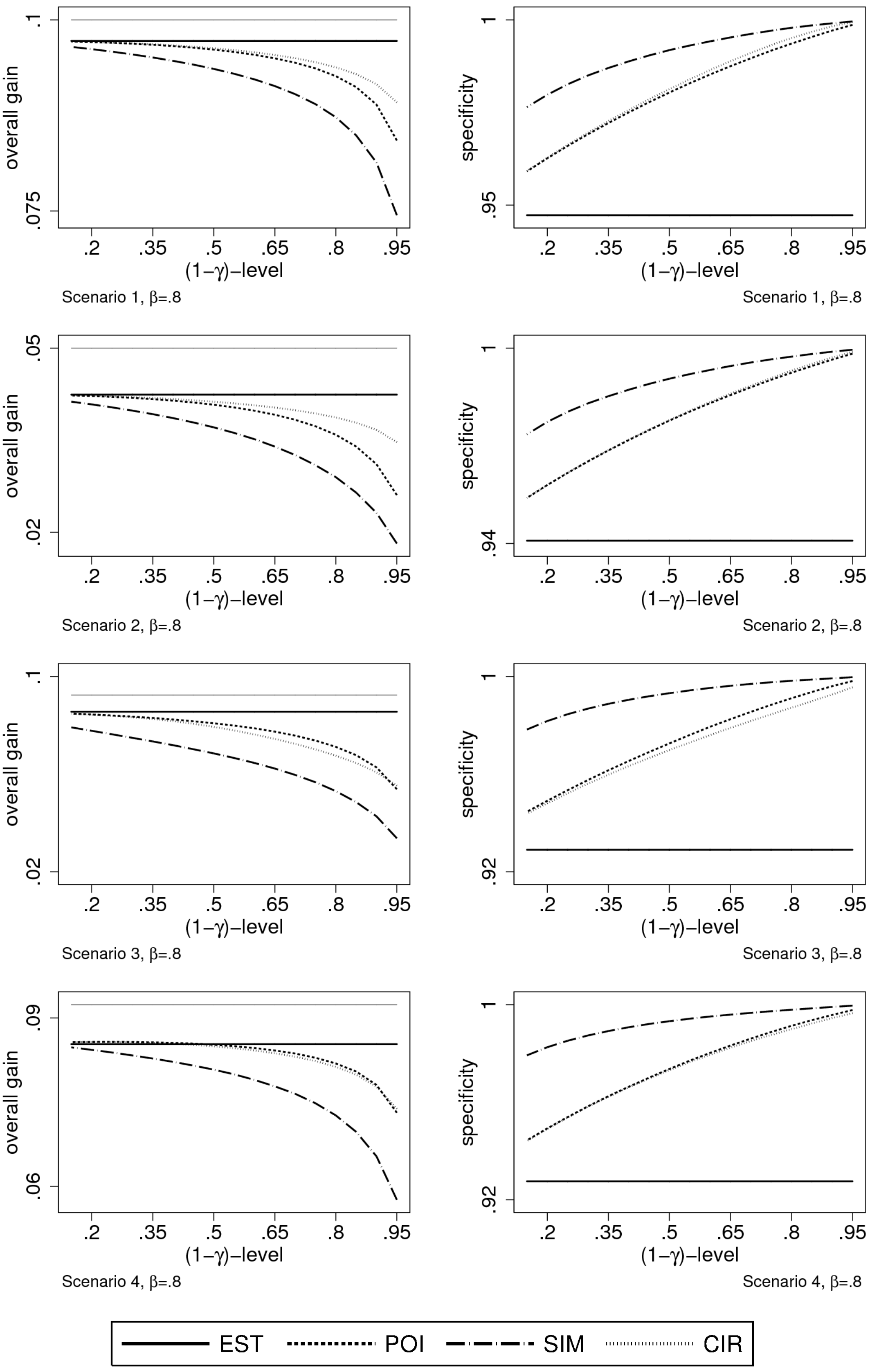

Figure Additional File 6c: Simulation results of overall gain and specificity for $\beta = 0.8$ in dependence of the $(1 - \gamma)$-level. Shown are all four scenarios individually. The first two scenarios are analysed using a linear model, the latter two using a quadratic model. For the overall gain, the thin grey line indicates the maximally possible overall gain.

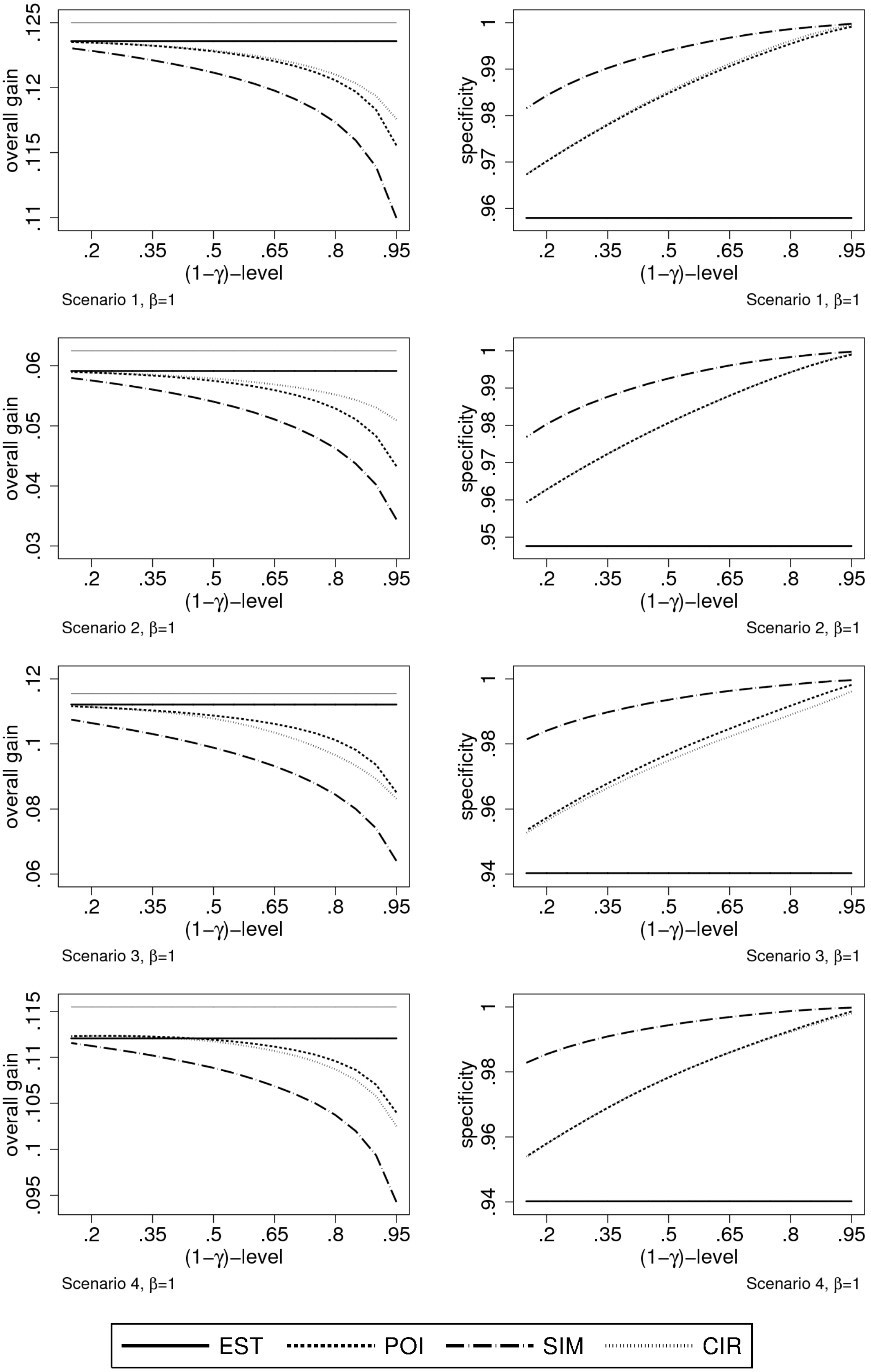

Figure Additional File 6d: Simulation results of overall gain and specificity for $\beta = 1$ in dependence of the $(1-\gamma)$-level. Shown are all four scenarios individually. The first two scenarios are analysed using a linear model, the latter two using a quadratic model. For the overall gain, the thin grey line indicates the maximally possible overall gain.

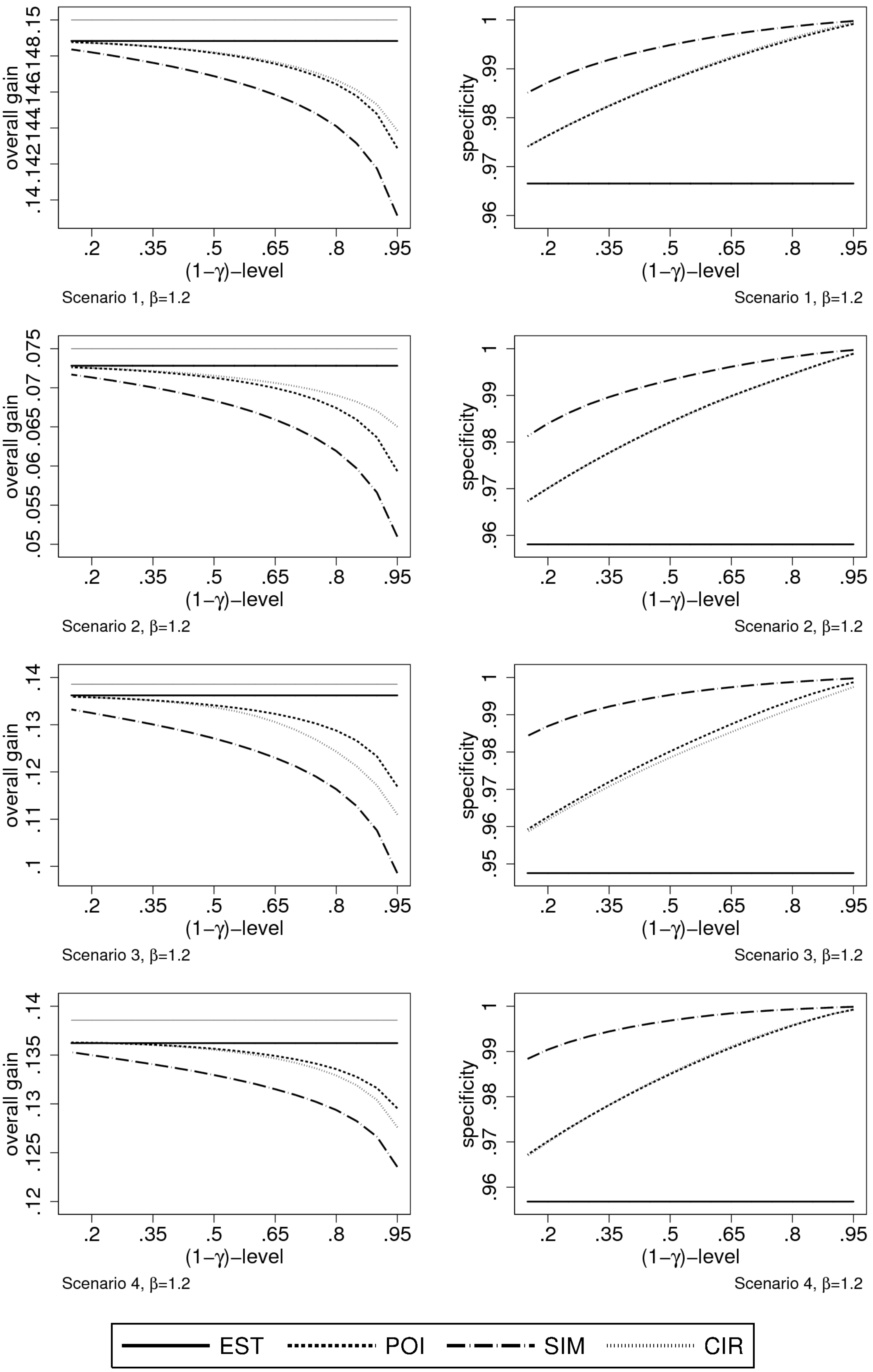

Figure Additional File 6e: Simulation results of overall gain and specificity for $\beta = 1.2$ in dependence of the $(1-\gamma)$-level. Shown are all four scenarios individually. The first two scenarios are analysed using a linear model, the latter two using a quadratic model. For the overall gain, the thin grey line indicates the maximally possible overall gain.
